# Commentary on: Self-Reported Breast Implant Illness: The Contribution of Systemic Illnesses and Other Factors to Patient Symptoms

**DOI:** 10.1093/asjof/ojad038

**Published:** 2023-04-13

**Authors:** Patricia McGuire

See the Original Article here.

Breast implant illness (BII) is a term used to describe a wide variety of systemic symptoms that patients attribute to their breast implants (Video). Over 100 symptoms have been reported in no specific configuration and there are currently no diagnostic criteria, this is a diagnosis of exclusion.^1^ Many of these patients have had extensive medical evaluations with no abnormal laboratory or physical findings to explain their symptoms and request implant removal as a last resort. Other patients will research their symptoms on the internet and may come across breast implant illness websites or social media groups. These patients may present requesting removal of their implants and capsules without an appropriate medical evaluation of their symptoms.^2^ As board certified plastic surgeons, we must practice evidence-based medicine and operate on medically stable patients with appropriate indications and with full informed consent.

The author in this study, “Self-Reported Breast Implant Illness: The Contribution of Systemic Illnesses and Other Factors to Patient Symptoms,”^3^ used the PubMed (National Institutes of Health, Bethesda, MD), Ovid (Wolters Kluwer, Alphen aan den Rijn, the Netherlands), and Google Scholar (Mountain View, CA) databases with a search strategy for the 6 most reported symptoms in patients with self-described breast implant illness and matched those to medical conditions with similar symptom patterns. They showed significant overlap between BII symptoms and fibromyalgia, chronic fatigue syndrome, autoimmune disorders, hypothyroidism, anxiety disorders, menopause and aging, medication side effects, and lifestyle choices such as smoking, marijuana use, and dietary choices, which are clearly presented in the tables. The author suggests patients should have a full medical evaluation prior to undergoing surgery.

The author references the paper by Glicksman et al which showed partial symptom improvement after implant removal in the majority of patients with BII in a prospective blinded study with controls, including a mastopexy cohort, who have never had breast implants.^4^ The author states that the control mastopexy cohort in that paper reported an increase in symptoms, such as anxiety, fatigue, and brain fog. I suspect the author's point in that statement is that the symptoms experienced by patients with BII are not uncommon symptoms even in women without breast implants, rather than that the mastopexy cohort showed an increase in symptoms. Patients in both the mastopexy and non-BII implant cohort in the Glicksman paper did report similar symptoms, but at levels statistically lower than the BII cohort and the mastopexy cohort symptom reporting did not change through the study follow-up.

Although there is evidence that patients with self-described breast implant illness will experience at least partial symptom improvement after implant removal, it is important to rule out other potential causes for symptoms prior to surgery. I thank the author for this paper and the important message that we need to address our patient's concerns and request for implant removal, but not without ensuring an appropriate medical evaluation prior to surgery.

## Supplementary Material

ojad038_Supplementary_Data

## References

[1] Magnusson MR, Cooter RD, Rakhorst H, McGuire PA, Adams WP, Deva AK. Breast implant illness: a way forward. Plastic Reconstr Surg. 2019;143(3S A Review of Breast Implant-Associated Anaplastic Large Cell Lymphoma):74S–81S. doi: 10.1097/PRS.0000000000005573 30817559

[2] McGuire P, Clauw DJ, Hammer J, Haws M, Adams WP. A practical guide to managing patients with systemic symptoms and breast implants. Aesthet Surg J. 2022;44(4):397–404. doi: 10.1093/asj/sjab375 PMC8922689 34687293

[3] Bresnick S. Self-reported breast implant illness: the contribution of systemic illnesses and other factors to patient symptoms. Aesthet Surg J Open Forum. 2023;5:1–9. doi: 10.1093/asjof/ojad030 PMC10111281 37082331

[4] Glicksman C, McGuire P, Kadin M, et al Impact of capsulectomy type on post-explantation systemic symptom improvement: findings from the ASERF systemic symptoms in women biospecimen analysis study. Aesthet Surg J. 2022;42(7):809–819. doi: 10.1093/asj/sjab417 34915566 PMC9208825

